# Three-dimensional analysis of posttreatment tooth movements despite bonded retainers: part I—upper jaw

**DOI:** 10.1007/s00056-024-00545-y

**Published:** 2024-08-27

**Authors:** Katharina Klaus, Tobias Kleinert, Sabine Ruf

**Affiliations:** 1 Department of Orthodontics, Justus Liebig University Giessen, Schlangenzahl 14, 35392 Giessen, Germany; 2 Private Practice, Balingen, Germany

**Keywords:** Bonded lingual retainer, Upper bonded retainer, Orthodontic retention, Retainer complications, Posttreatment malocclusion, Festsitzende Lingualretainer, Oberkiefer-Kleberetainer, Kieferorthopädische Retention, Retainerkomplikationen, Fehlstellung nach Behandlung

## Abstract

**Purpose:**

Adverse side effects of fixed retainers in terms of unwanted tooth movements have been described for both the upper and lower jaw, but data about the extent and movement patterns for the maxilla are scarce. The purpose of the present retrospective case–control study was to analyze the amount and direction of unwanted tooth movements despite upper bonded retainers as well as to analyze possible predisposing pretreatment- and treatment-related factors.

**Methods:**

Plaster casts of 1026 patients who completed orthodontic treatment and a subsequent retention phase of 2 years were screened for unintentional tooth movements. The study group comprised 57 patients with visually obvious tooth movements in the upper jaw, while 57 randomly selected patients without visible tooth movements served as control group. For all patients, plaster casts after debonding of multibracket appliance (T1) and after supervised retention (T2) were digitized, and superimposed digitally using a stable palatal reference area. Thereafter, translational and rotational movements were measured in all three planes of space. Pretreatment- and treatment-related factors of the study and control groups were compared by χ^2^ test, exact Fisher test, Mann–Whitney U test, and the T‑test for independent samples.

**Results:**

The mean translational movements ranged between 0 and 0.6 mm and the average rotational movements between 0 and 1.3°. Large individual movements up to 2.7 mm translation and 15.9° rotation were seen. A movement pattern around the Y‑ and Z‑axis with an opposite rotational peak at the canines (“upper twist effect”) was identified. Compared to the control group, patients of the study group showed a significantly smaller intercanine width pretreatment. Also, study group patients presented a larger intercanine expansion and a slightly larger overjet reduction during treatment, and were more often affected by retainer bonding site detachments and wire fractures, but without reaching statistically significance.

**Conclusion:**

Upper bonded retainers show a similar unwanted movement pattern (“twist effect”) like the one described for mandibular retainers.

## Abstract (de)

**Zielsetzung:**

Komplikationen festsitzender Retainer im Sinne unerwünschter Zahnbewegungen wurden bereits für Ober- und Unterkiefer beschrieben, jedoch liegen speziell für den Oberkiefer nur wenige Daten zu Ausmaß und möglichen Bewegungsmustern vor. Ziel dieser retrospektiven Fall-Kontroll-Studie war es, das Ausmaß und die Richtung unerwünschter Zahnbewegungen trotz festsitzender Retainer im Oberkiefer zu analysieren sowie mögliche beeinflussende prätherapeutische und therapeutische Faktoren zu identifizieren.

**Material und Methode:**

Kieferorthopädische Studienmodelle von 1062 Patienten, die ihre kieferorthopädische Behandlung inklusive 2‑jähriger Retentionszeit abgeschlossen hatten, wurden hinsichtlich unerwünschter Zahnbewegungen gesichtet. Die Studiengruppe umfasste 57 Patienten mit visuell sichtbaren Zahnbewegungen, 57 zufällig ausgewählte Patienten ohne sichtbare Zahnbewegungen dienten als Kontrollgruppe. Für alle Patienten wurden die kieferorthopädischen Studienmodelle nach Entfernung der Multibracketapparatur (T1) sowie nach 2‑jähriger Retentionszeit (T2) eingescannt und digital über eine stabile palatinale Referenzstruktur überlagert. Im Anschluss wurden translatorische und rotatorische Zahnbewegungen in allen drei Dimensionen vermessen. Mögliche prätherapeutische und therapeutische Faktoren der Studien- und Kontrollgruppe wurden mittels χ^2^-Test, exaktem Fisher-Test, Mann-Whitney-U-Test sowie dem T‑Test für unabhängige Stichproben verglichen.

**Ergebnisse:**

Die durchschnittlichen translatorischen Bewegungen lagen zwischen 0 und 0,6 mm, während die durchschnittlichen rotatorischen Bewegungen 0 bis 1,3° betrugen. Große individuelle Bewegungen bis zu 2,7 mm Translation und 15,9° Rotation wurden beobachtet. Ein Bewegungsmuster um die Y‑ und Z‑Achse mit maximaler gegensätzlicher Bewegung der Eckzähne („Twist-Effekt im Oberkiefer“) konnte identifiziert werden. Im Vergleich mit der Kontrollgruppe wiesen die Patienten der Studiengruppe eine signifikant geringere prätherapeutische intercanine Distanz auf. Außerdem zeigten die Patienten der Studiengruppe während der Behandlung eine größere intercanine Expansion sowie eine größere Overjetreduktion und waren häufiger von Klebestellenverlusten im Bereich des Retainers sowie von Drahtbrüchen betroffen, jedoch ohne statistische Signifikanz.

**Schlussfolgerung:**

Festsitzende Retainer im Oberkiefer zeigen ähnliche Bewegungsmuster („Twist-Effekt“), wie sie bereits für den Unterkiefer beschrieben wurden.

## Introduction

After orthodontic treatment, the achieved results have to be stabilized using removable or fixed retainers [19, 20]. The greatest advantage of fixed retention is its independence from patient cooperation [21, 25, 27]. Questionnaire-based survey studies among orthodontists in the United States, Australia, and New Zealand as well as in Northern Europe revealed that fixed lingual retainers bonded to all six anterior teeth are the retention of choice for the lower jaw [3, 17, 21, 25, 27, 33, 37]. For the upper jaw, removable Hawley retainers and vacuum-formed retainers are preferred by orthodontists in the United States and Australia/New Zealand, whereas upper fixed retainers are routinely used by only 2.4–11% of the practitioners [25, 33, 37]. In contrast, in the Netherlands and Switzerland, despite of small exceptions, the majority of orthodontists preferred bonded retainers for both jaws. Factors affecting the choice for vs. against fixed upper retainers were the pretreatment situation, specific treatments (e.g., extractions of permanent teeth or arch expansion), posttreatment occlusion, oral hygiene, and deep bite cases [17, 27].

While canine-to-canine retainers bonded to all six anterior teeth are standard in the mandible, maxillary bonded retainers range from two to six bonding sites involving the central incisors only (1–1), central and lateral incisors (2–2) or all six anterior teeth including the upper canines (3–3). Especially the posttreatment occlusion and a posttreatment deep bite seem to affect the practitioners’ choice regarding the extension of the upper bonded retainers [17, 27, 28, 37].

Adverse side effects in terms of tooth movement despite bonded retainers are reported by 56.7–94.7% of orthodontists [3, 17, 21]. Such movements are classified as newly developed malocclusions rather than relapse, because of a lack of similarity with the pretreatment malocclusion [10, 15, 16, 26]. A recently published systematic review used the term “wire syndrome” for those complications and found more studies describing affected mandibular jaws than maxillary jaws [8]. Regarding mandibular fixed retainers, a prevalence of 1.1–5% is reported [10, 16, 26]. Flexible spiral wire retainers are estimated to be more often responsible than thick stainless-steel retainers bonded only to the canines [10]. To our knowledge, only five case reports/case series describing unexpected tooth movements despite bonded retainers in the upper jaw are available [1, 7, 12, 24, 30]. They comprised all possible extensions of upper retainers ranging from 1–1 to 3–3 and described proclination of central incisors, spacing between the incisors, angulation/torque changes of central and lateral incisors, or increased buccal torque of the terminal teeth within the retained segment [1, 7, 12, 24, 30]. Besides these case reports, only two retrospective studies mentioned maxillary retainer complications in larger patient cohorts [13, 14]. The first study compared 44 patients with tooth movements in the upper and/or lower jaw to 43 patients without complications and found maxillary retainers to be affected more often than mandibular retainers. Upper teeth showed more extrusive and protrusive movements than lower teeth, while the amount of movements was similar in both jaws. Nevertheless, only translational (bodily) tooth movements were analyzed, while rotational (tipping) tooth movements were not evaluated [13]. The second study analyzed the spontaneous recovery tooth movements after debonding of affected retainers over a period of 14 weeks. More pronounced recovery movements were found in the upper jaw [14]. All in all, solid information about maxillary bonded retainer complications is still scarce, and information about possible predisposing factors is lacking.

Therefore, the aim of the present retrospective case–control study was to investigate the amount and direction of translational and rotational posttreatment tooth movements despite upper bonded retainers as well as to analyze possible predisposing factors.

## Materials and methods

Approval for the present retrospective investigation was granted by the ethics committee of the medical faculty of the Justus Liebig University Giessen, Germany (number 71/18).

### Patients

All patients who completed active orthodontic treatment plus a subsequent supervised retention period of approximately 2 years at the Department of Orthodontics of the Justus Liebig University Giessen, Germany, over 11 consecutive years (2005–2015) were assessed. The inclusion criteria were

- Pretreatment class I malocclusion according to Angle [2],
- Normally inclined or proclined incisors pretreatment,
- Multibracket appliance treatment in both jaws (Tip-Edge Plus, TP Orthodontics, La Porte, IN, USA),
- Fixed cuspid-to-cuspid retainers, bonded on all six anterior teeth (3–3) in the upper and lower jaw,
- Intact plaster casts with good quality from three different timepoints: pretreatment (T0), after debonding of the multibracket appliance (T1), and after the supervised retention period of approximately 2 years (T2),
- Intact 3–3 retainers in situ at T2, and
- No active orthodontic intervention/retreatment between T1 and T2 for any reason.

Patients were excluded if

- They had additional removable retainers,
- Teeth in the anterior segment (canines, lateral or central incisors) were missing, e.g., due to aplasia or dental trauma,
- They received adhesive or prosthetic restorations of the canines, lateral, or central incisors between T1 and T2,
- Their plaster casts were not suitable for digitization and superimposition due to gypsum fractures or obviously distorted teeth, or
- Their plaster casts at T1 exhibited a pronounced gingival hyperplasia which hindered the assessment of tooth position.

### Retention protocol

The retention protocol has already been described elsewhere [13]. In brief, all retainers were manufactured by orthodontically experienced dental technicians using a 0.018 inch 6‑strand coaxial wire (Dentaflex®, Dentaurum GmbH & Co. KG, Ispringen, Germany) and bonded by the orthodontists and residents of the department following the direct bonding method described by Zachrisson and Büyükyilmaz [38] using Transbond XT or Transbond LR (3M Unitek, Monrovia, CA, USA) according to the manufacturers’ instructions. Retainer control appointments were scheduled at 3 and 6 months after retainer bonding and biannually thereafter. In the cases with retainer debonding or wire breakage, patients were advised to make an emergency appointment as soon as possible. Detached bonding sites were rebonded passively. Wire breakages were repaired chairside by adaptation and bonding of a second bridging wire (same material as the original retainer wire) to the two teeth beside the wire breakage.

### Methods

**Fig. 1:**
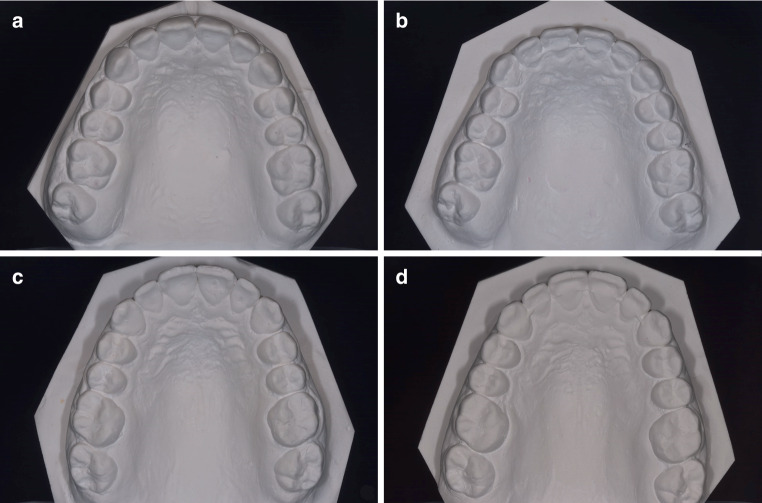
Exemplary cases of visual inspection for assignment to the study (**a**, **b**) and control (**c**, **d**) group, respectively. **a** T1 model of study group patient, **b** T2 model of the same patient. Please note the offset between teeth 13 and 14, **c** T1 model of control group patient, **d** T2 model of the same patient Exemplarische Fälle visueller Inspektion für die Zuteilung zur Studien- (**a**, **b**) bzw. zur Kontrollgruppe (**c**, **d**). **a** T1-Modell eines Studiengruppenpatienten, **b** T2-Modell desselben Patienten. Zu beachten ist der Versatz zwischen den Zähnen 13 und 14, **c** T1-Modell eines Kontrollgruppenpatienten, **d** T2-Modell desselben Patienten

**Fig. 2:**
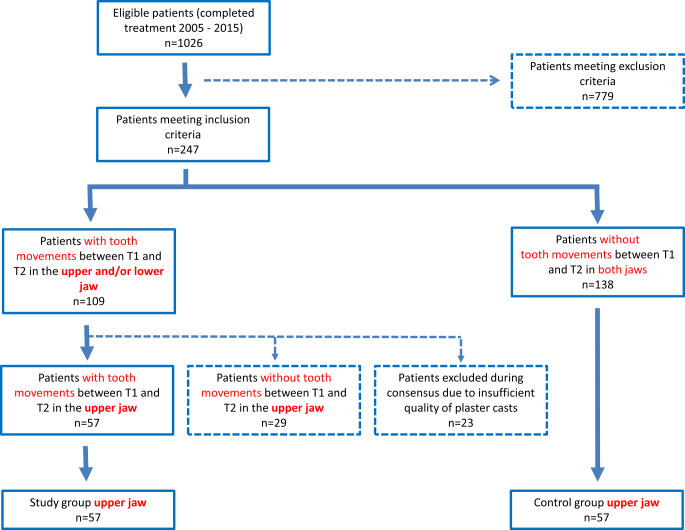
Flowchart of study population Flussdiagramm zur Studienpopulation

The methods used for case identification and model digitization have also been described elsewhere [13]. The plaster casts of all patients who met the inclusion criteria were carefully assessed by one investigator (T.K.). All T1 and T2 models were visually inspected comparing the tooth positions of the canines, lateral and central incisors between the two time points. All suspicious casts were additionally judged by two experienced orthodontists (K.K., S.R.). In uncertain cases, the incisal edges and marginal ridges were colored using a soft pencil to reach consensus. Figure 1 shows the plaster casts of one exemplary case assigned to the study group and one assigned to the control group based on visual inspection. A flow chart illustrating the study population is given in Fig. 2. All patients with agreement among all authors regarding tooth movements in the upper jaw between T1 and T2 were assigned to the study group (*n* = 57). For the control group, an equal number of patients (*n* = 57) was randomly chosen out of the cohort of patients without visible tooth movements in both jaws (Fig. 2).

**Fig. 3:**
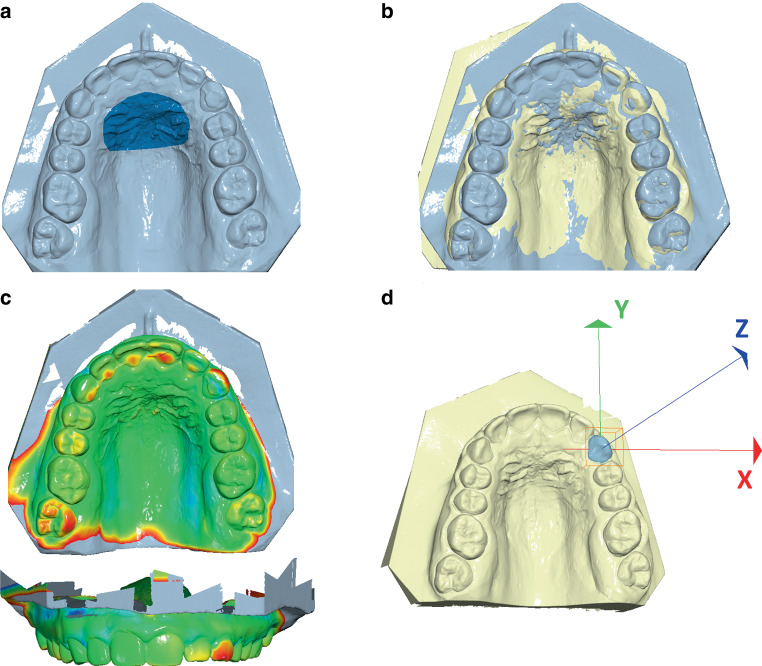
**a** The palatal reference area used for superimposition of the digital models according to Stucki and Gkantidis [31], **b** Superimposed models from time points T1 (after multibracket appliance removal, *yellow*) and T2 (after 2 years of supervised fixed retention, *light blue*), **c** Colormap indicating the deviations based on the T2 model ranging between +1.5 mm (*red*) and −1.5 mm (*blue*), **d** Orientation of axes for measurement of tooth movements: X‑axis (*red*), Y‑axis (*green*), and Z‑axis (*blue*) **a** Der palatinale Referenzbereich für die digitale Modellüberlagerung gemäß Stucki und Gkantidis [31], **b** Überlagerte Modelle der Zeitpunkte T1 (nach Entfernung der Multibracketapparatur, *gelb*) und T2 (nach 2 Jahren überwachter festsitzender Retention, *hellblau*), **c** Farbige Abweichungsdarstellung projiziert auf das T2-Modell, reichend von +1,5 mm (*rot*) bis −1,5 mm (*blau*), **d** Achsenorientierung für die Messung der Zahnbewegungen: X‑Achse (*rot*), Y‑Achse (*grün*) und Z‑Achse (*blau*)

Subsequently, all upper plaster casts of the study and control groups (T1, T2) were digitized using a desktop scanner (OrthoXScan, Dentaurum, Ispringen, Germany) and saved as Standard Tesselation Language (STL) files. The digitized casts from T1 and T2 were further imported into the Viewbox 4 software (Version 4.1.0.6 BETA, dHAL software, Kifissia, Greece) and superimposed using the stable area of the hard palate around the third rugae. The lateral margins of the stable area were set at more than 5 mm from the gingival margins (Fig. 3a; [31]). The software performs a local best-fit superimposition using the implementation of the iterative closest point algorithm (ICP) [4]. The ICP setting was 100% estimated overlap of meshes, matching point to plane, exact nearest neighbor search, 100% point sampling, 50 iterations (Fig. 3b; [34, 35]). After superimposition, a color-coded distance map was created indicating deviations of +1.5 mm (red) and −1.5 mm (blue) of the superimposed models. The colormap displayed the movement tendencies of the teeth within the retained segment (Fig. 3c). Furthermore, the movements of all teeth showing a color deviation were measured in all three planes of space, as described elsewhere [34, 35]. In brief, following the former cast superimposition, each tooth of the T2 model was superimposed on the respective tooth of the T1 model, using the superimposition settings described above. For each measurement, the origin of the coordinate system was placed at the crown centroid of the respective tooth on the T1 model [39]. The X‑axis (red) was parallel to the occlusal plane and perpendicular to the median palatine suture, the Y‑axis (green) was parallel to the occlusal plane and parallel to the midline and the Z‑axis (blue) was perpendicular to the occlusal plane (Fig. 3d). Movements along the X‑axis represent lateral translational movements (positive: right, negative: left), those along the Y‑axis anterior–posterior translational movements (positive: protrusion, negative: retrusion) and those along the Z‑axis vertical translational movements (positive: extrusion, negative: intrusion) and were measured in millimeters (mm). Rotational movements around the X‑axis represent inclination changes in the anteroposterior direction (positive: proclination, negative: retroclination), those around the Y‑axis inclination changes in the lateral direction (positive: right, negative: left) and those around the Z‑axis rotational changes (positive: counterclockwise, negative: clockwise) and were recorded in degrees (°). All superimpositions and measurements were obtained by one trained investigator (T.K.). To assess the intraobserver reliability of superimpositions and measurements, the whole procedure was repeated for 10 randomly selected patients after 4 weeks.

For all patients (study and control group), the following data were obtained from the patients’ records: sex and age of the patients at time points T0, T1, and T2; existence of pretreatment habits (sucking habit, lip or tongue dysfunction, tongue thrust swallowing, bruxism, mouth breathing); pretreatment cephalometric mandibular plane angle (ML/NSL) and upper incisor inclination angle (IsL/NA); extraction of premolars during active treatment; overjet and incisal relationship (interincisal contact, incisal overlap without interincisal contact, open bite) as well as intercanine distance at T0, T1, and T2 measured on the plaster casts. Furthermore, retainer complications between T1 and T2 in terms of bonding site detachments or wire breakages were recorded. All plaster cast measurements (overjet, intercanine distance) were undertaken with a caliper (Zürcher Modell, Karl Hammacher GmbH, Solingen, Germany) to the nearest 0.5 mm by one single investigator (T.K.). Due to the fact that only T1 and T2 plaster casts had been digitized, but the respective measurements were undertaken at T0, T1, and T2 casts, overjet, and intercanine distance were measured manually.

### Statistical analysis

The intraobserver reliability of repeated digital superimposition and measurement of tooth movements was assessed calculating intraclass correlation coefficients (ICCs).

Besides descriptive analyses, the categorical variables of the study and control group were compared by the χ^2^ test and the exact Fisher test. Due to the fact that not all numeric variables were distributed normally (Kolmogorov–Smirnov test, Shapiro–Wilk test), the Mann–Whitney U test and the T‑test for independent samples were applied. Statistics were carried out by a certified medical biostatistician using the software SPSS for windows, version 25.0 (IBM Corp., Armonk, NY, USA). The level of significance was set at *p* < 0.05.

## Results

The study group consisted of 57 patients (19 males, 38 females) with a mean pretreatment age of 12.49 ± 2.17 years. Active orthodontic treatment (T0–T1) of the study group patients lasted 3.14 ± 1.03 years, while the duration of supervised retention was 2.08 ± 0.35 years. The control group comprised 57 patients (18 males, 37 females) with an average age of 12.11 ± 1.7 at T0, a mean active treatment time of 3.17 ± 1.18 years, and a supervised retention period of 2.08 ± 0.37 years. Sex, age, and treatment time were not significantly different between the groups.

### Tooth movements

The intraclass correlation coefficient (ICC) for repeated digital superimpositions was 0.951, while the ICC for the measurement of tooth movements was 0.978, both indicating excellent intraobserver reliability.

Three-dimensional (3D) superimposition confirmed the visually noticeable tooth movements in the study group but also revealed tooth movements in the control group which had not become obvious during visual inspection of the plaster casts. Pooling the prevalence of translational and rotational tooth movements detected by 3D superimposition along all axes, the highest movement prevalence of the study group was found for tooth 21 (98.2%), followed by teeth 11 and 12 (each 89.5%) and 22 (87.7%). The canines had the lowest prevalence (13: 84.2%, 23: 80.7%). Despite the fact that the prevalence in the control group was much lower, the distribution regarding the different teeth was comparable with the study group (Table 1).

**Table 1:** Absolute number of stable and affected patients per movement direction and group; mean values, standard deviations (SD), minimum, median, and maximum values as well as 25th and 75th percentiles of tooth movement measured by three-dimensional superimposition per tooth in the study and control group Absolute Anzahl stabiler und betroffener Patienten pro Bewegungsrichtung und Gruppe; Mittelwerte, Standardabweichungen (SD), Minima, Mediane und Maxima sowie 25. und 75. Perzentile der mittels dreidimensionaler Überlagerung gemessenen Zahnbewegungen pro Zahn in der Studien- und der Kontrollgruppe

		Study group						Control group					
		13	12	11	21	22	23	13	12	11	21	22	23
Translational along X‑axis [mm]	Stable [*n*]	9	6	6	1	7	11	34	31	25	24	30	32
	Lateral right (+) [*n*]	21	26	31	28	25	24	12	7	15	17	16	10
	Lateral left (−) [*n*]	27	25	20	28	25	22	11	19	17	16	11	15
	Mean	−0.04	−0.05	0.44	−0.04	0.01	0.03	−0.01	0.06	−0.02	0	0.01	0.01
	SD	0.37	0.38	0.55	0.54	0.31	0.26	0.28	0.20	0.22	0.23	0.15	0.19
	Minimum	−1.18	−1.19	−1.27	−2.75	−0.88	−0.48	−0.84	−0.72	−0.84	−0.84	−0.45	−0.35
	25th	−0.24	−0.22	0	−0.22	−0.17	−0.16	0	−0.12	−0.08	−0.03	0	0
	Median	0	0	0.53	0	0	0	0	0	0	0	0	0
	75th	0.10	0.15	0.75	0.28	0.16	0.21	0	0	0.04	0.07	0.01	0
	Maximum	0.81	0.89	0.83	1.09	0.60	0.69	1.00	0.39	0.44	0.63	0.60	0.64
Rotational around X‑axis [°]	Stable [*n*]	9	6	6	1	7	11	35	31	25	24	30	32
	Proclination (+) [*n*]	13	12	21	28	10	13	9	6	15	14	16	10
	Retroclination (−) [*n*]	35	39	30	28	40	33	13	20	17	19	11	15
	Mean	−1.36	−2.74	−0.90	−0.61	−2.44	−0.78	−0.12	−0.64	−0.09	−0.08	0.01	0.01
	SD	3.44	4.31	3.89	4.19	3.45	2.91	1.13	1.65	1.90	2.29	0.15	0.19
	Minimum	−9.31	−15.96	−11.46	−9.87	−11.20	−8.23	−3.35	−6.76	−5.53	−4.43	−8.27	−3.82
	25th	−3.46	−4.90	−2.89	−3.50	−4.73	−2.32	0	−1.33	−0.36	−0.74	0	0
	Median	−0.51	−2.63	−0.19	0	−2.52	−0.52	0	0	0	0	0	0
	75th	0	0	1.49	1.87	0	0	0	0	0.18	0	0.01	0
	Maximum	4.99	6.94	7.80	8.79	5.77	6.14	5.30	3.06	4.88	11.96	13.19	4.38
Translational along Y‑axis [mm]	Stable [*n*]	9	6	6	1	7	11	34	31	25	24	29	32
	Protrusion (+) [*n*]	35	44	41	40	39	32	10	24	31	32	26	22
	Retrusion (−) [*n*]	13	7	10	16	11	14	3	2	1	1	2	3
	Mean	0.29	0.45	0.44	0.42	0.40	0.23	0.22	0.26	0.37	0.37	0.28	0.21
	SD	0.59	0.53	0.55	0.66	0.54	0.56	0.38	0.36	0.41	0.40	0.42	0.44
	Minimum	−2.15	−1.45	−0.93	−2.08	−1.10	−1.49	−0.41	−0.18	−0.30	−0.15	−0.36	−0.59
	25th	0	0.06	0	−0.04	0	0	0	0	0	0	0	0
	Median	0.31	0.56	0.53	0.51	0.36	0.20	0	0	0.28	0.28	0	0
	75th	0.70	0.75	0.75	0.90	0.78	0.62	0.55	0.61	0.70	0.66	0.60	0.30
	Maximum	1.60	1.94	2.01	1.72	1.63	1.58	1.19	1.26	1.16	1.42	1.87	1.98
Rotational around Y‑axis [°]	Stable [*n*]	9	6	6	1	7	11	35	31	25	24	29	32
	Lateral right (+) [*n*]	20	29	29	32	33	26	8	17	17	11	12	20
	Lateral left (−) [*n*]	28	22	22	24	17	20	14	9	15	22	16	5
	Mean	−1.12	−0.28	0.29	0.18	0.75	1.18	−0.29	0.43	0	−0.27	−0.45	0.51
	SD	3.76	3.67	1.86	2.00	2.90	3.36	2.25	2.05	1.13	1.32	2.04	1.60
	Minimum	−14.47	−7.92	−4.88	−4.14	−8.14	−3.63	−10.99	−6.96	−2.64	−4.24	−7.09	−4.98
	25th	−2.9	−2.72	−0.64	−1.06	−0.86	−0.96	0	0	−0.11	−0.74	−0.66	0
	Median	0	0.10	0.01	0.79	0.24	0	0	0	0	0	0	0
	75th	0.49	1.70	1.37	1.42	2.73	2.04	0	0.58	0.29	0	0	1.10
	Maximum	5.97	11.76	5.47	5.33	7.36	13.60	5.45	7.16	2.94	4.19	3.11	5.14
Translational along Z‑axis [mm]	Stable [*n*]	9	6	6	1	7	11	35	31	25	24	29	32
	Extrusion (+) [*n*]	44	44	41	47	40	38	21	25	31	31	25	24
	Intrusion (−) [*n*]	4	7	10	9	10	8	1	1	1	2	3	1
	Mean	0.66	0.56	0.44	0.47	0.39	0.47	0.34	0.40	0.44	0.47	0.44	0.37
	SD	0.58	0.57	0.64	0.68	0.55	0.69	0.52	0.55	0.51	0.55	0.58	0.53
	Minimum	−0.64	−0.65	−1.30	−1.49	−1.34	−1.23	−0.06	−0.30	−0.06	−0.10	−0.05	−0.24
	25th	0.27	0.13	0	0.09	0	0	0	0	0	0	0	0
	Median	0.63	0.46	0.44	0.52	0.42	0.47	0	0	0.21	0.12	0	0
	75th	1.05	0.91	0.78	0.93	0.73	0.85	0.61	0.89	0.90	0.90	1.04	0.78
	Maximum	2.15	2.27	2.10	1.71	1.55	2.18	1.76	1.66	1.70	1.84	1.73	1.79
Rotational around Z‑axis [°]	Stable [*n*]	9	6	6	1	7	11	34	31	25	24	29	32
	ccw (+) [*n*]	34	26	29	28	27	16	12	13	15	21	10	13
	cw (−) [*n*]	14	25	22	28	23	30	11	13	17	12	18	12
	Mean	0.99	0.15	0.29	−0.21	0.05	−1.30	0.15	0.05	−0.22	0.22	−0.29	−0.18
	SD	3.48	3.62	2.19	2.00	2.77	3.21	1.57	1.69	1.2	1.43	1.87	1.91
	Minimum	−9.16	−11.62	−5.80	−5.96	−7.45	−11.37	−5.82	−6.60	−3.45	−5.39	−6.86	−6.39
	25th	0	−1.35	−0.36	−1.57	−1.67	−3.27	0	0	−0.48	0	−0.35	0
	Median	1.09	0	0.07	0	0	−0.50	0	0	0	0	0	0
	75th	3.08	2.1	1.76	1.14	1.66	0.24	0	0	0.02	0.75	0	0
	Maximum	8.34	7.48	4.64	4.98	8.19	4.36	4.16	7.48	3.26	3.45	5.59	8.05

*mm* millimeter, *ccw* counterclockwise, *cw* clockwise

Regarding the absolute amount of tooth movements pooled for all teeth, the study group showed significantly (*p* < 0.001) higher movements than the control group irrespective of the direction.

### Tooth movements along and around the X-axis

**Fig. 4:**
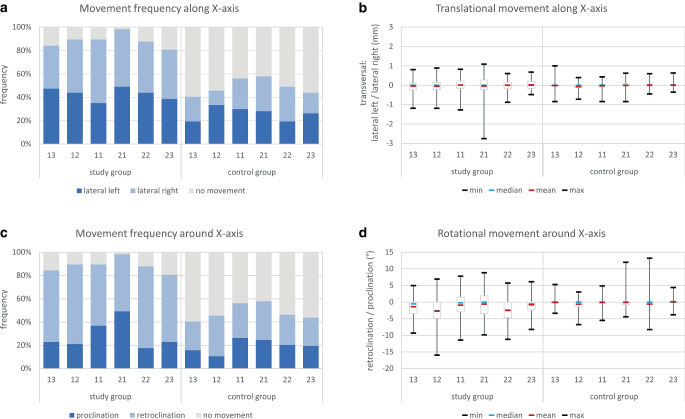
Tooth movements along (**a**, **b**) and around (**c**, **d**) the X‑axis. **a** Frequency of translational tooth movements, **b** amount of translational tooth movements in mm, **c** frequency of rotational tooth movements, and **d** amount of rotational tooth movements in ° Zahnbewegungen entlang der (**a**, **b**) und um die (**c**, **d**) X‑Achse. **a** Häufigkeit der translatorischen Bewegungen, **b** Ausmaß der translatorischen Zahnbewegungen in mm, **c** Häufigkeit der rotatorischen Zahnbewegungen und **d** Ausmaß der rotatorischen Zahnbewegungen in °

For translational movements along the X‑axis (Fig. 4a; Table 1), the frequency of right and left tooth translations were nearly equally distributed between the different teeth. The mean and median amount of lateral translational movements varied slightly around zero, with maximum values of about 1 mm except for tooth 21, which showed a maximum translation of 2.7 mm to the left (Fig. 4b; Table 1).

For rotational movements around the X‑axis (Fig. 4c; Table 1), all teeth of the study group showed a higher frequency of retroclination, except tooth 21, in which the frequency of proclination and retroclination was equal. The mean amount of retroclination ranged between 0.6 and 2.7°, whereas outliers up to 15.9° retroclination and 8.7° proclination were seen. Among the tooth movements detected in the control group, retroclination was also more frequent, whereby the centrals and the left lateral incisors had a more equal distribution between pro- and retroclination compared to the other teeth. In the control group, mean and median amount of rotational movements ranged around zero, but substantially outliers in both directions were also seen (Fig. 4d; Table 1).

### Tooth movements along and around the Y-axis

**Fig. 5:**
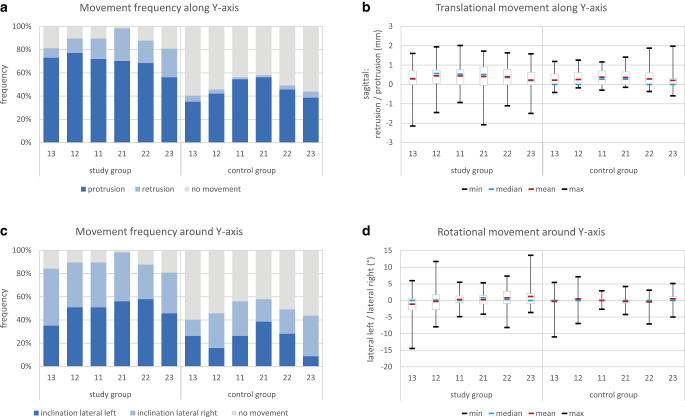
Tooth movements along (**a**, **b**) and around (**c**, **d**) the Y‑axis. **a** Frequency of translational tooth movements, **b** amount of translational tooth movements in mm, **c** frequency of rotational tooth movements, and **d** amount of rotational tooth movements in ° Zahnbewegungen entlang der (**a**, **b**) und um die (**c**, **d**) Y‑Achse. **a** Häufigkeit der translatorischen Bewegungen, **b** Ausmaß der translatorischen Zahnbewegungen in mm, **c** Häufigkeit der rotatorischen Zahnbewegungen und **d** Ausmaß der rotatorischen Zahnbewegungen in °

For translational movements along the Y‑axis (Fig. 5a; Table 1), protrusive movements prevailed in both the study and control groups. The mean and median amount in the study group ranged between 0.2 and 0.5 mm. In the control group, the mean amount was 0.2–0.3 mm for all teeth. Maximum protrusive translation was similar in the study and control group and amounted up to 2 mm. In contrast, the maximum amount of retrusion was much higher in the study group and reached up to 2 mm for teeth 13 and 21 (Fig. 5b; Table 1).

For rotational movements around the Y‑axis (Fig. 5c; Table 1), a distinct movement pattern in the study group could be seen. The mean amount, the interquartile range, and the maximum of rotational movements around the Y‑axis showed a gradual side shift from a left inclination for the right canine to a right inclination for the left canine. While the mean amount of canine tipping was 1.1° in the respective direction, the maximum inclination was 14.5° to the left for 13 and 13.5° to the right for 23. This indicates a distal inclination of all retained teeth which was most pronounced at the terminal teeth and less pronounced at the central teeth of the retained segment. For the control group, no similar movement pattern was detectable (Fig. 5d; Table 1).

### Tooth movements along and around the Z-axis

**Fig. 6:**
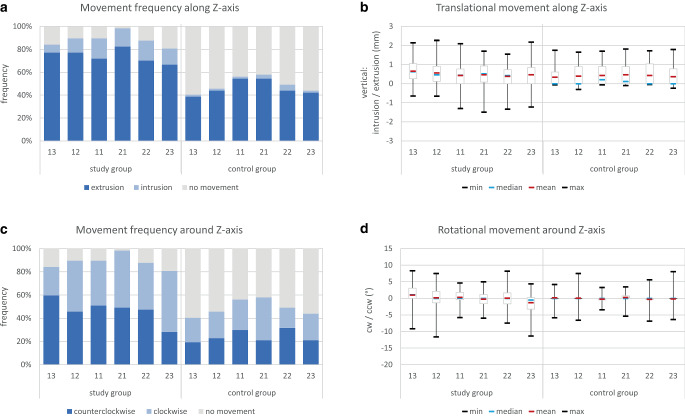
Tooth movements along (**a**, **b**) and around (**c**, **d**) the Z‑axis. **a** Frequency of translational tooth movements, **b** amount of translational tooth movements in mm, **c** frequency of rotational tooth movements, and **d** amount of rotational tooth movements in °; *cw* clockwise, *ccw* counterclockwise Zahnbewegungen entlang der (**a**, **b**) und um die (**c**, **d**) Z‑Achse. **a** Häufigkeit der translatorischen Bewegungen, **b** Ausmaß der translatorischen Zahnbewegungen in mm, **c** Häufigkeit der rotatorischen Zahnbewegungen und **d** Ausmaß der rotatorischen Zahnbewegungen in °; *cw* im Uhrzeigersinn, *ccw* gegen den Uhrzeigersinn

For translational movements along the Z‑axis (Fig. 6a; Table 1), a much higher frequency (66.7–82.5%) of extrusive compared to intrusive movements (7.0–17.5%) was seen in the study group. Also in the control group, extrusions prevailed, but were observed at a much lower frequency (38.6–54.4%), whereas intrusive movements were almost absent (1.8–5.3%). The mean amount of extrusion ranged between 0.4 and 0.6 mm for the study group. The control group presented similar averages for all teeth. The same was true for the maximum extrusive values, which were only slightly higher in the study group (1.5–2.3 mm) compared to the control group (1.6–1.8 mm). Intrusive outliers, on the contrary, were only seen in the study group and ranged between 0.6 and 1.5 mm (Fig. 6b; Table 1).

For rotational movements around the Z‑axis (Fig. 6c; Table 1), the frequency of both directions (clockwise/counterclockwise) was nearly equally distributed in both the study and the control group except for both canines in the study group: while 13 was more frequently affected by counterclockwise rotation, the opposite was true for 23 which presented clockwise rotation more often. For the study group, the mean amount of movement around the Z‑axis ranged between 1° counterclockwise rotation for tooth 13 to 1.3° clockwise rotation for tooth 23 and alike the distribution of interquartile ranges reflected a side shift of the retained segment. In contrast to the similar movement pattern around the Y‑axis, the maximum outliers did not confirm this pattern for the Z‑axis. Nevertheless, a tendency for contrary rotations at the terminal ends of the retained segment was visible (Fig. 6d; Table 1).

### Possible predisposing factors

Neither the pretreatment mandibular plane angle (study group: 33.6°, control group: 32.4°, *p* = 0.235) nor the pretreatment degree of upper incisor proclination (study: 22.5°, control: 22.4°, *p* = 0.976) displayed a significant intergroup difference. The overjet reduction during treatment was slightly larger in the study group (1.62 ± 1.55 mm) compared to the control group (1.53 ± 1.93 mm), but this difference was not statistically significant (*p* = 0.333). The pretreatment intercanine distance in the study group (33.2 ± 2.51 mm) was smaller than in the control group (34.8 ± 3.04 mm) and that difference was found to be statistically significant (*p* = 0.047). During treatment, the intercanine distance was expanded by 1.7 ± 2.85 mm in the study group, while the control group only presented an expansion of 0.4 ± 2.3 mm. Nevertheless, this difference did not reach statistical significance (*p* = 0.061, Table 2). The same was true for cases with premolar extractions during treatment. Habits that existed prior to treatment as well as the interincisal relationship showed also no significant difference (Table 3).

**Table 2:** Mean values and standard deviations (SD) of metric pretreatment- and treatment-related variables in the study and control group Mittelwerte und Standardabweichungen (SD) der metrischen prätherapeutischen und therapeutischen Variablen in der Studien- und der Kontrollgruppe

	Study group		Control group		*p*-value
	Mean	SD	Mean	SD	
Age at T0 [years]	12.49	2.17	12.11	1.7	0.304
Age at T1 [years]	15.63	2.28	15.28	1.57	0.493
Age at T2 [years]	17.71	2.18	17.36	1.55	0.405
Duration of treatment [years]	3.14	1.03	3.17	1.18	0.962
Duration of retention phase [years]	2.08	0.35	2.08	0.37	0.770
ML/NSL [°]	33.6	6.03	32.4	4.53	0.235
IsL/NA [°]	22.5	6.16	22.43	6.25	0.976
Overjet at T0 [mm]	3.75	1.66	3.6	1.87	0.468
Overjet at T1 [mm]	2.13	0.83	2.07	0.82	0.702
Overjet at T2 [mm]	2.42	1.03	2.18	0.79	0.182
Overjet reduction during treatment (T0–T1) [mm]	1.62	1.55	1.53	1.93	0.333
Intercanine distance T0 [mm]	33.22	2.51	34.82	3.04	0.047*
Intercanine distance T1 [mm]	35.02	1.66	35.21	1.76	0.374
Intercanine distance T2 [mm]	35.42	1.65	35.38	1.74	0.918
Expansion of intercanine distance during treatment (T0–T1) [mm]	1.69	2.85	0.4	2.3	0.061

*Statistically significant difference

*ML/NSL* mandibular plane angle, *IsL/NA* incisor inclination angle, *mm* millimeters

**Table 3:** Absolute and relative frequency of premolar extractions, pretreatment habits, and interincisal relationship for the study and control group Absolute und relative Häufigkeit von Prämolarenextraktionen, prätherapeutischen Habits und der interinzisalen Relation in der Studien- und der Kontrollgruppe

	Study group		Control group		*p*-value
	n	%	n	%	
*Premolars extracted during treatment*					
Yes	18	31.6	20	35.1	0.691
No	39	68.4	37	64.9	
*Habits pretreatment*					
Yes	26	45.6	24	42.1	0.706
No	31	54.4	33	57.9	
*Interincisal relationship T0*					
Interincisal contact	46	80.7	50	87.7	0.304
Overlap without contact	10	17.5	7	12.3	0.430
Open bite	1	1.8	0	0.0	0.315
*Interincisal relationship T1*					
Interincisal contact	48	84.2	43	75.4	0.243
Overlap without contact	9	15.8	14	24.6	0.243
Open bite	0	0.0	0	0.0	1
*Interincisal relationship T2*					
Interincisal contact	41	71.9	48	84.2	0.113
Overlap without contact	15	26.3	7	12.3	0.058
Open bite	1	1.8	2	3.5	0.558

Besides tooth 23, all teeth of the study group were affected more often by detachment of bonding sites between T1 and T2. This difference was statistically significant for tooth 21 only (Table 4). Wire breakages were also more frequently observed in the study group (24.6%) compared to the control group (10.6%), but no statistically significant difference was detectable for this finding (Table 5).

**Table 4:** Absolute and relative frequency of bonding site detachments during supervised retention period for the study and control group Absolute und relative Häufigkeit von Klebestellenverlusten während der überwachten Retentionszeit für die Studien- und die Kontrollgruppe

Detachments of bonding;sites (T1–T2)	Study group		Control group		*p*-value
	*n*	%	*n*	%	
13	13	22.8	7	12.3	0.329
12	17	29.8	7	12.3	0.097
11	13	22.8	5	8.8	0.059
21	12	21.1	4	7.0	0.043*
22	13	22.8	6	10.5	0.153
23	12	21.1	12	21.1	1

*Statistically significant differences

**Table 5:** Absolute and relative frequency of wire breakages during supervised retention period for the study and control group Absolute und relative Häufigkeit von Drahtbrüchen während der überwachten Retentionszeit für die Studien- und die Kontrollgruppe

Wire breakages (T1–T2)	Study group		Control group		*p*-value
	*n*	%	*n*	%	
Interdental 13/12	2	3.5	2	3.5	1
Interdental 12/11	3	5.3	1	1.8	0.618
Interdental 11/21	2	3.5	1	1.8	1
Interdental 21/22	3	5.3	0	0.0	0.243
Interdental 22/23	4	7.0	2	3.5	0.679

## Discussion

To our knowledge, this is the first study investigating both translational and rotational unwanted tooth movements despite bonded maxillary retainers in situ. Furthermore, to date no investigation dealing with such retainer complications has compared movements of affected and unaffected individuals. Despite some case reports and small case series [1, 7, 12, 24, 30], only two retrospective studies have mentioned unwanted tooth movements in the upper jaw [13, 14]. In concordance with the present study results, a predominance of extrusive and protrusive movements with a comparable amount was reported [13].

Regarding translational movements, the comparatively high prevalence and amount of extrusive and protrusive movements in the study as well as in the control group are noteworthy. Albeit the prevalence was still much higher in the study group, particularly the mean amount and the maximum values of extrusion and protrusion were comparable between the groups. Especially for extrusive movements, it has to be questioned whether the measured values are truly reflecting unwanted tooth movements rather than settling tendencies occurring after debonding of the multibracket appliance, slight relapse tendencies of deep overbite cases, or extrusive movements which are related to individual vertical growth. Studies investigating relapse of deep bites over a comparable period of time as in the present population (2–2.3 years) showed an average increase in overbite of about 0.5–1.0 mm [5, 6, 18], which corresponds well to the mean extrusion of 0.3–0.5 mm found in the present study, assuming that a deep bite relapse is caused half by extrusive movements of the upper and half by extrusion of the lower anterior teeth. In addition, a vertical downward movement of the palatal rugae and the incisors in growing patients has to be suspected, since the palatal height increases even during adulthood [22, 32].

The most interesting movement patterns observed in the present study population were the rotational changes around the Y‑ and Z‑axis, where a shift to the opposite side with maximum amounts of rotation at the terminal teeth of the retained segment was seen. In studies investigating unwanted tooth movements in lower bonded retainers, the so-called “twist effect” has already been described, indicating an opposite torque direction of the contralateral canines [16, 36]. The present study is, however, the first to present comparable effects for upper bonded retainers. Unfortunately, the underlying mechanisms and reasons for this complication are still unknown. The fact that in some cases the terminal teeth of the retained segment are most severely affected and express opposite movements could possibly be associated with the structure of the flexible spiral wire retainer and its winding/unwinding direction [16, 36], which is also supported by actual in vitro results [29].

Regarding the analysis of possible predisposing factors, the pretreatment intercanine distance was significantly smaller in the study group resulting in an insignificantly larger expansion than in the control group. A similar observation was described by Wolf et al. [36] analyzing lower bonded retainers. In addition, patients in the present study group were affected more often by bonding site detachments during the supervised retention period. Even though all patients were advised to immediately fix an appointment in case of bonding site detachments, it cannot be ruled out that some detachments were not noticed by the patients and that the teeth remained detached until the next routine appointment. This could have led to minor tooth displacements, clinically unnoticed neither by the patient nor the practitioner, but visible by the 3D superimposition or which were noticed clinically, but after consensus with the patient were too small to justify retreatment.

The present study has some limitations. First, as the study was designed as a retrospective case–control study, no sample size calculation was carried out a priori. Second, the strictly chosen inclusion and exclusion criteria led to the exclusion of many patients especially due to the required pretreatment class I malocclusion and because of compromised plaster casts. The latter is inevitably associated with the retrospective nature of the investigation, as manufacturing and storage of casts was not driven by the intention to produce and maintain standards high enough for digitization and superimposition. The total number of patients could have been increased by additionally including patients with pretreatment class II or class III occlusal relationships. Third, not all retainers were bonded by one single operator but instead by different orthodontists and residents of the department. On the one hand, the selection of cases treated by one single operator would have led to a further decrease in potential study subjects. On the other hand, several authors discussed that an iatrogenic activation of the retainer during bonding could lead to unwanted tooth movements [1, 10, 16, 23, 30, 36]. To keep the influence of this factor as small as possible, all retainers were bonded following the same protocol. Hence, an iatrogenic activation during bonding cannot totally be ruled out. In such cases, however, tooth movements should have occurred within a few weeks after bonding. The onset of unwanted tooth movements is still unclear. Kucera and Marek [16] found the majority of events occurring during 0–6 years in retention, and thus one could argue whether the present observation period of 2 years was appropriate. Nevertheless, there are comparable studies analyzing lower retainers based on follow-up periods of 6 months to 3 years [10, 13, 36]; therefore, the chosen period of 2 years seems acceptable. A longer follow-up period would be a desirable goal for further research.

Fourth, initial screening of unwanted tooth movements was based on visual inspection of T1 and T2 plaster casts, which is of course a subjective evaluation, and demonstrated limitations because the 3D superimposition revealed tooth movements in the control group also. Due to the fact that digital superimposition as well as measurement of tooth movements is still very time consuming, the visual inspection added by consensus rounds with two experienced orthodontists seemed to be an acceptable compromise reflecting a level of clinical relevance. In addition, to date no scientifically validated thresholds or cut-off values for the identification of unwanted tooth movements are established. Hence, even a digital superimposition and measurement of all casts meeting the inclusion criteria would have required a somewhat subjective determination of threshold values. Furthermore, during clinical retention control appointments, the teeth are visually inspected by the orthodontist, too. Therefore, tooth movements have to be visible to the extent to gain the attention of the patient and the orthodontist. The fact that also some movements of reasonable extent were digitally detected in the control group emphasizes the difficulty to visually assess especially vertical movements and accentuates the accuracy of digital measurements.

Finally, a potential limitation could be seen in the method of digital superimposition. Notwithstanding that no intraoral area is absolutely stable due to growth and remodeling [9, 11], the area used in the present study was found to provide reliable outcomes [31]. However, the translational vertical measurements should be interpreted with caution due to the ongoing increase of palatal depth during adolescence and adulthood [23, 32].

Because of the small number of studies addressing complications of unwanted tooth movements despite fixed bonded maxillary retainers and their still unknown etiology, further research is needed to identify patients at risk for such complications and in order to reduce the need for orthodontic retreatment.

## Conclusions

The present study showed that translational and rotational tooth movements in all three planes of space occur despite bonded retainers in the maxilla. Although the mean translational movements ranged between 0 and 0.6 mm and the average rotational movements between 0 and 1.3°, large individual movements up to 2.7 mm translation to the right and 15.9° retroclination were seen in the study group. As already described for mandibular retainers, a movement pattern around the Y‑ and Z‑axis with an opposite rotational peak at the canines was identified (“upper twist effect”). Patients with reduced intercanine width and also greater intercanine expansion during treatment could be at higher risk and should be monitored meticulously. Ideally, upper bonded retainers should be carefully supervised as long as they are left in place.

## Data Availability

The datasets supporting the conclusions of this article are available from the corresponding author on reasonable request.
